# Novel gene similar to nitrite reductase (NO forming) plays potentially important role in the latency of tuberculosis

**DOI:** 10.1038/s41598-021-99346-1

**Published:** 2021-10-06

**Authors:** Sonia Agrawal, Suwarna Gample, Amar Yeware, Dhiman Sarkar

**Affiliations:** 1 Organic Chemistry Division, CSIR-National Chemical Laboratory, Dr. Homi Bhabha Road, Pune, Maharashtra 411008 India; 2 Academy of Scientific and Innovative Research (AcSIR), Ghaziabad, 201002 India

**Keywords:** Biochemistry, Biological techniques, Genetics, Microbiology, Molecular biology

## Abstract

The development of the latent phenotype of *Mycobacterium tuberculosis* (Mtb) in the human lungs is the major hurdle to eradicate Tuberculosis. We recently reported that exposure to nitrite (10 mM) for six days under in vitro aerobic conditions completely transforms the bacilli into a viable but non-cultivable phenotype. Herein, we show that nitrite (beyond 5 mM) treated Mtb produces nitric oxide (NO) within the cell in a dose-dependent manner. Our search for the conserved sequence of NO synthesizing enzyme in the bacterial system identified *MRA2164* and *MRA0854* genes, of which the former was found to be significantly up regulated after nitrite exposure. In addition, the purified recombinant *MRA2164* protein shows significant nitrite dependent NO synthesizing activity. The knockdown of the *MRA2164* gene at mRNA level expression resulted in a significantly reduced NO level compared to the wild type bacilli with a simultaneous return of its replicative capability. Therefore, this study first time reports that nitrite induces dormancy in Mtb cells through induced expression of the *MRA2164* gene and productions of NO as a mechanism for maintaining non-replicative stage in Mtb. This observation could help to control the Tuberculosis disease, especially the latent phenotype of the bacilli.

## Introduction

*Mycobacterium tuberculosis* (Mtb) infects alveolar macrophages in the lung as a primary target and causes pulmonary Tuberculosis (TB) disease in humans. In 2019, about 1.4 million people died in the world due to TB^1^. Mtb could be found in either replicating (as an active state) or remain in a non-replicating (dormant or latent) form to create a major hurdle in eradicating the disease^2^. Several reports showed that mycobacteria could survive in the hostile environment in the presence of reactive oxygen species (ROS) or reactive nitrosative intermediates (RNI) stress in the lungs^3–5^. But there is no report about their quantification in the lungs to understand the level of Mtb bacilli exposure to such toxic chemicals. Although Mtb has evolved multiple mechanisms to evade toxic effects of ROS or RNI by engaging different enzymes such as catalase, peroxidase, and alkyl hydroperoxide reductase, little is known about their role in the pathogenesis of the bacilli^6^. Recent studies on different *Mycobacterium spp*. point toward the involvement of these oxidative metabolites in shifting the replicative status of the bacilli^7–10^. Superoxide is found as pro-growth and NO as pro- latency or a growth regulator^7,11^. It is also suggested that NO interacts with superoxide to yield peroxynitrite radical which rearranges to produce nitrate^12,13^. While nitrate is a stable RNI that can be utilized as an alternative respiratory substrate by Mtb during hypoxia, acid as well as RNI stress, otherwise as nitrogen source during growth^14–16^. The *narK2*, a nitrite/nitrate antiporter expressed in the bacterial membrane, is up regulated under hypoxic conditions^17,18^.

The pulmonary TB patients were earlier reported to release an increased extent of NO in exhaled air as well as NO metabolites in urine^19,20^. Besides elevated expression of inducible nitric oxide synthase (*iNOS),* the presence of nitro-tyrosine (product of tyrosine and peroxynitrite) are detected in granuloma that demonstrates its increased production during tuberculous infection^21^. NO produced in the host macrophages could be converted into nitrite by dimeric hemoglobin (HbN) or with an oxygenated aqueous medium^15,22,23^. It was also observed that the exposure of Mtb bacilli to NO induce dormancy phenotype coupled with increased expression of the dormancy regulon related genes^11^. NO synthesized from macrophages is widely known to act as a transiently active signal molecule that raises doubt about its continued presence as an inducer of latency for prolong period. In addition, there has been no such study done to focus on the nitrite concentration build up at the site of the infected tissue. Interestingly, nitrite has recently been shown as a robust inducer of dormancy in Mtb from in vitro experiments^24^. Also the presence of a significantly high amount of nitrite was observed in collected sputum samples from TB infected patients^25^.

Here, we report that nitrite is reduced to NO by Mtb *MRA2164* gene product, with very similar functional characteristics of bacterial *nirK* and its possible role in Mtb, validated with fluorescence microscopy, the enzymatic activity of the recombinant protein as well as knock down experiments.

## Results

### Nitrite dependent intracellular NO synthesis in *Mycobacterium tuberculosis*

**Figure 1:**
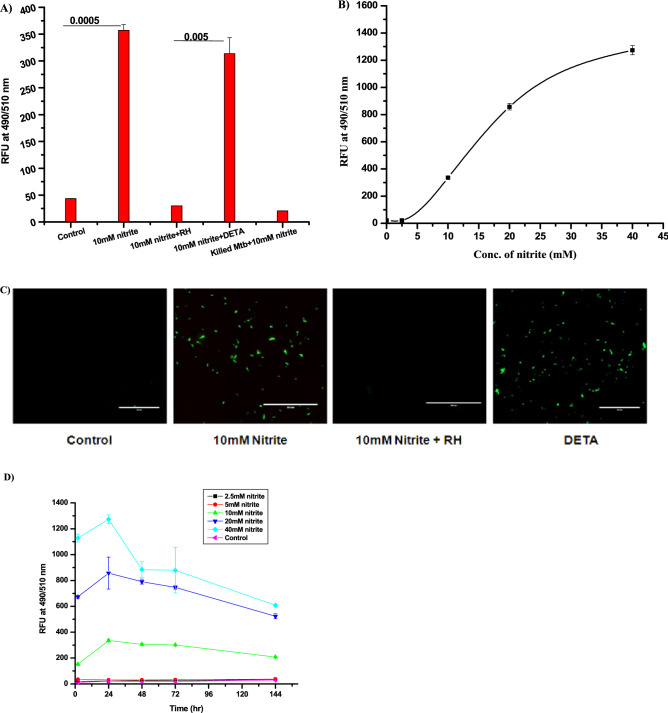
NO production in *Mycobacterium tuberculosis* cells in the presence of nitrite: Log phase Mtb cells were treated with (**A**) nitrite (10 mM), nitrite (10 mM) along with rutin hydrate (1 µM) and DETA NO (50 µM), killed Mtb (10 mM of nitrite), (**B**) In presence of different nitrite concentration (**C**) Fluorescence microscopy image of nitrite treated Mtb cells captured by EVOS microscope as mention in “Materials and Methods section” (Scale bar 50 μm). (**D**) Time kinetics of NO production in Mtb cells at different concentration of nitrite with (black filled square) 2.5 mM, (black filled circle) 5 mM, (black filled triangle) 10 mM (black filled inverted triangle) 20 mM, (black filled daimond) 40 mM nitrite and, (black filled left side triangle) untreated as control respectively. NO production in Mtb cells was detected by using DAF2DA dye as relative fluorescence unit at 490/510 nm wavelength. The data shown are representative as a mean of three independent experiments ± SD.

As NO is already known as an inducer of dormancy in mycobacteria coupled with the reported fact that it could be produced from nitrite as well in several other bacterial species, we checked the production of NO in Mtb cells in the presence of nitrite in the medium^11,26^. In order to do this, Mtb cells were exposed to a cell permeable DAF2-DA dye, of which DAF2 specifically reacts with NO^27,28^ to produce green fluorescence of triazofluorescein complex^29^. A significant difference in fluorescence intensity was observed when Mtb cells are exposed to (356 ± 11 relative fluorescent units (RFU)) nitrite (10 mM) compared to (43 ± 2 RFU) untreated control Mtb cells (Fig. 1A). Apart from Mtb culture, we have checked fluorescence intensity by using dead Mtb cells, *E. coli* and *M. smegmatis* (*Msm*) culture of same optical density (OD_600_ nm) in place of Mtb cells under identical conditions (Fig. 1A & Fig. S2). Dead Mtb cells and *E. coli* culture did not show any significant increase of intensity as observed in the blank (10 mM nitrite in medium without Mtb cells). But *M. smegmatis* cells with nitrite have shown fluorescence intensity similar to Mtb cells. This indicates that both *Mycobacterium spp*. are using a similar mechanism of nitrite dependent NO production, which is absent in *E. coli*.

There was no difference between fluorescence intensities of DAF2 DA observed when nitrite was exposed to different buffers under acidic and alkaline conditions, suggesting the involvement of intracellular protein/s in this conversion. The increased fluorescence level in nitrite treated Mtb cells was completely abolished (29 ± 1 RFU) in the presence of 1 µM of rutin hydrate (RH) (a NO scavenger). Inversely, treatment of Mtb cells with 50 µM of Diethylenetriamine (DETA), a NO adduct as a positive control showed an enhanced level (313 ± 15 RFU) of fluorescence which indicated that the increase in intracellular fluorescence developed after exposure with nitrite was due to the generation of NO inside the Mtb cells^11^. Furthermore, exposure of Mtb cells to increased concentrations of nitrite followed a sigmoidal pattern of increase in intracellular DAF2 fluorescence (Fig. 1B). The fluorescence microscopic studies under similar conditions also suggested that NO is significantly produced within Mtb cells in the presence of nitrite (Fig. 1C). Further detection of NO at different time points after exposure of Mtb cells to nitrite clearly indicated that NO producing capacity is relatively rapidly increased to attain a peak within 24 h, which then slowly decreases to a minimum level (Fig. 1D). Altogether, the results confirmed that nitrite exposed Mtb cells are produce intracellular NO and attain peak level within ~ 24 h.

### *MRA2164* and *MRA 0854* as possible nitrite reductase in *Mycobacterium tuberculosis*

**Figure 2:**
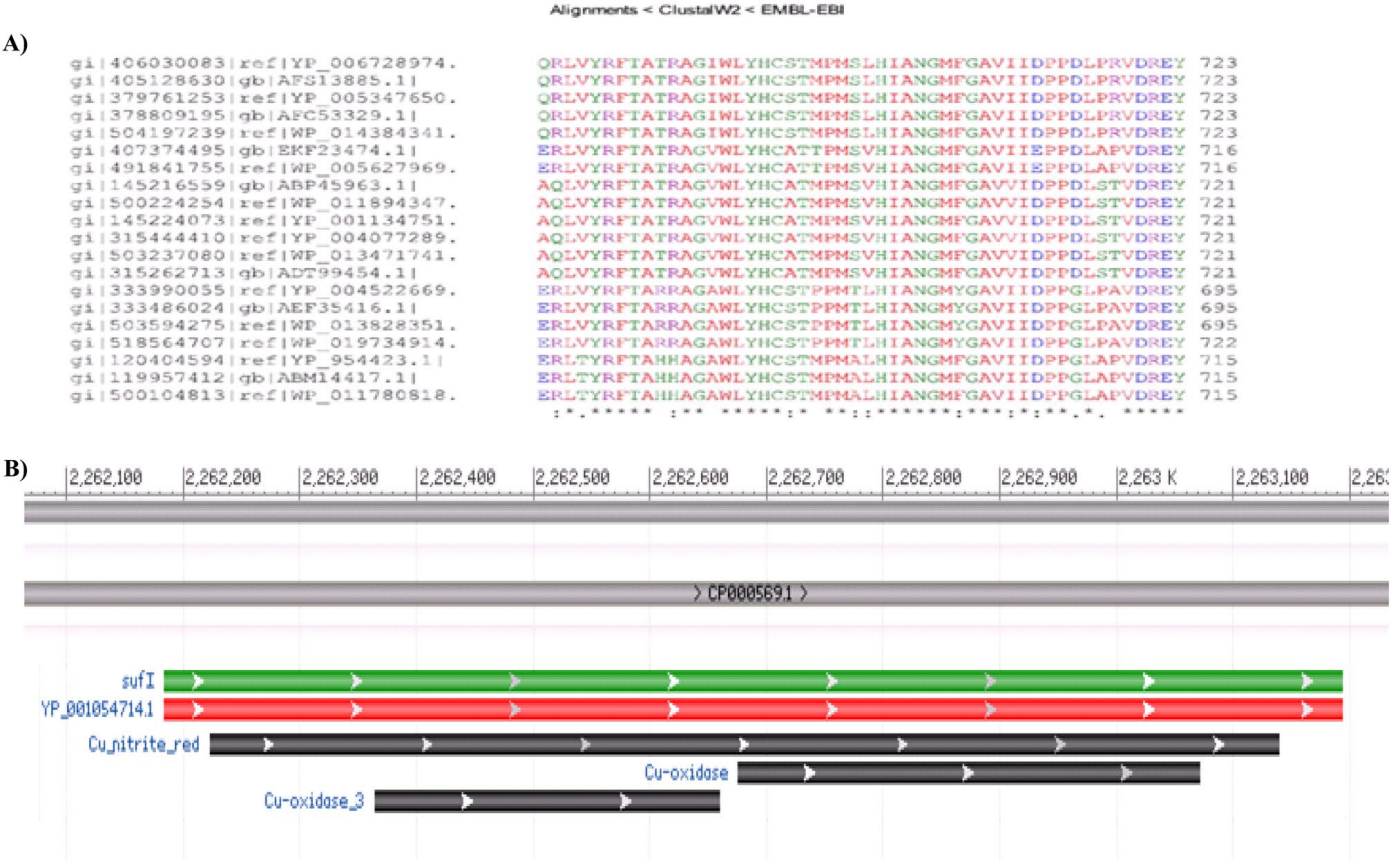
(**A**–**B**) Identification of conserved domains of genes in *Mycobacterium tuberculosis* (**A**) Conserved sequences obtained after alignment of gene sequences coding for nitrite reductase of different bacteria by ClustalW2 software (http://www.ebi.ac.uk/Tools/msa/clustalo/). (**B**) Gene sequences containing conserved domain Cu-oxidase and Cu-oxidase-3 of Mtb H37Ra nitrite reductase. Data was extracted from CD search software.

Although assimilatory type nitrite reductase (*nirBD*) is present in Mtb, there is no report about the presence of NO synthesizing enzyme in any *Mycobacterium spp*. There are two classes of nitrite reductases (*nir*) present in the bacterial system, one class consists of a multi heme enzymes which reduces nitrate to a variety of products; and the others are copper containing enzyme which carries out single electron transfer to produce NO from nitrite^30^. Here, the conserved regions were first identified from the nitrite reductase (*nirK* or NO forming) gene by using gene sequences from other bacteria which are reported to have this functional gene (Fig. 2A). The conserved sequence contains the copper oxidase 2, 3 and 4 conserved domains (CDs) detected in identified bacterial nitrite reductase (Fig. 2B). However, the number and type of CDs vary from one bacterial species to another. In some bacteria like *M. vanbaalenii*, nitrite reductase contains only one CDs, *i.e.,* copper oxidae-3; whereas, *Alcaligenes xylosoxidan*s gene contains two CDs, *i.e.,* copper oxidase 2 and 3^31^. Thus, based on the conserved sequence similarity, analyzed by clustalW2, the conserved domains of Cu-oxidase 2 and Cu-oxidase 4 are found to be present in *MRA0854* and *MRA2164* (Conserved hypothetical protein) genes of Mtb respectively.

### Expression of *MRA 2164* and *MRA0854* gene in *Mycobacterium tuberculosis*

**Figure 3:**
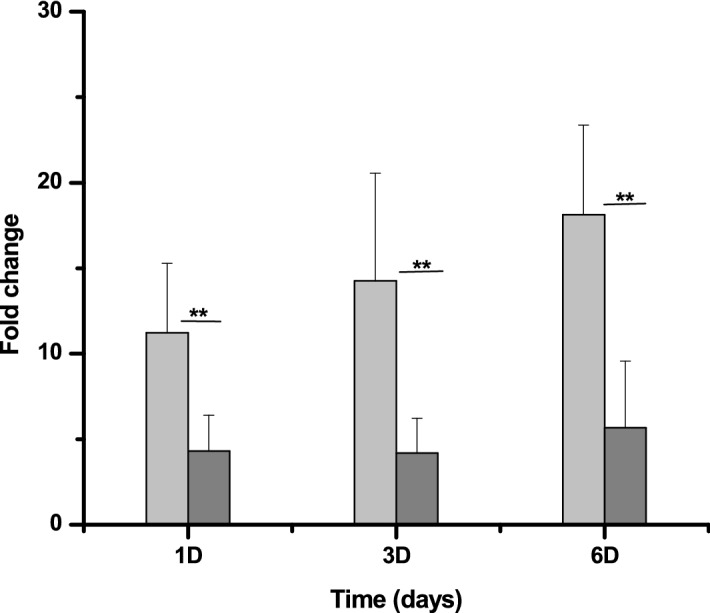
Quantification of *MRA2164* and *MRA0854* genes expression in nitrite treated *Mycobacterium tuberculosis* cells: The total RNA was extracted from untreated and nitrite (1, 3 and 6 days) treated Mtb cells and reverse transcribed into cDNA. Relative quantification done by using SYBR green based qPCR kit and fold change was calculated compared to untreated Mtb where *SigA* gene was used as a housekeeping reference. The light gray and gray color shows the fold change of *MRA2164* gene and *MRA0854* gene respectively. The data represented as the mean of three experiments ± SD (**P < 0.05).

The expression of the possible nitrite reductase genes (*MRA2164* and *MRA0854)* were checked by estimating their respective mRNA levels in nitrite treated Mtb cells using the q-PCR technique. It was observed that the expression of the *MRA2164* gene is steadily up regulated to attain 18.12 ± 5.24 fold compared to untreated control (*P* < 0.05) after 6 day of nitrite exposure to Mtb cells (Fig. 3). The *MRA0854* gene expression was neither increased to a significant level (5.66 ± 4.94 fold) nor consistent with time. So, we decided to pursue *MRA2164* as *nirK* for further characterizations.

### Characterization and functional analysis of recombinant *MRA2164 (nirK)* gene product

**Figure 4:**
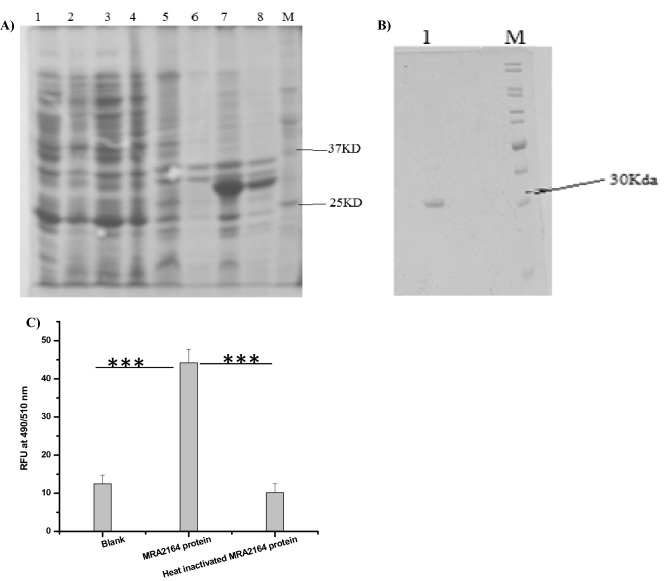
(**A**–**C**) Cloning, expression and purification of the *MRA2164* gene (**A**) SDS PAGE analysis of the recombinant: *MRA2164* protein Lanes 1–4 sequentially represent supernatants from *E. coli* Rosetta-gami, pET-28a as vector control, induced and uninduced of *MRA2164*, 5–8 are pellet from *E. coli* Rosetta-gami, vector control, induced and uninduced of *MRA2164* gene sequentially were separated by SDS/PAGE, (**B**) SDS-PAGE of the purified recombinant enzyme with Lane 1, purified *MRA2164* protein concentration of 4 µg; Lane M, Molecular weight markers are indicated at Right. (**C**) NO synthesizing activity of the pure protein in presence of nitrite as a substrate. More details are provided in the “Materials and Methods” section. The data represented as the mean of three experiments ± SD (***P < 0.01).

*MRA2164* sequence with His-tag was cloned into the pET28a vector (Fig. S3) and overexpressed in *E. coli* Rosetta-gami (Fig. 4A). The purity of the eluted protein was checked by SDS-PAGE analysis (Fig. 4B). It was observed as a single band of ~ 28 kDa from SDS-PAGE (Fig. 4B). The LC–MS analysis confirmed the protein as a product of *MRA2164* gene from Mtb. The TOF/TOF mass spectra analysis of the purified protein was compared with the genome database of Mtb H37Ra from UniProt and the protein band was identified as Polyphenol oxidase OS Mtb strain ATCC 25,177 H37Ra OX 419,947 GN yfiH PE 3 with a molecular weight of 26 kDa (identified as *MRA2164*) (Table 1) and peptide sequence was provided in Table 2.

**Table 1:** TOF/TOF mass spectra database results.

MW	PLGS score	Peptides	Peptides products	Theoretical	Digest peptides
25,948	21,137.69	55	584	20	14

**Table 2:** Peptides identified from recombinant *Mycobacterium tuberculosis MRA2164* protein.

Peptide sequence	Retention time	Overlapping sequence position	
		Start	End
(R)AGGVSAPPFDTFNLGDHVGDDPAAVAANR(A)	37.2971	28	56
(R)LAAAIGLPGNR(V)	30.37	59	69
(R)VVWMNQVHGDRVELVDQPR(N)	31.4467	70	88
(R)NTALDDTDGLVTATPR(L)	30.13	89	104
(R)LALAVVTADCVPVLMADAR(A)	43.6312	105	123
(R)DISALLGPAVSGR(N)	38.5591	158	170
(R)NYEVPAAMADEVEAALPGSR(T)	39.9091	171	190
(R)TTTAAGTPGVDLR(A)	25.9293	191	203
(R)AGIACQLR(D)	23.247	201	211
(R)DLGVESIDVDPR(C)	32.1036	212	223
(R)CTVADPTLFSHR(R)	28.9386	224	235
(T)VADPTLFSHR(R)	31.1381	226	235
(R)FASLVWME(-)	44.7715	243	250

**Figure 5:**
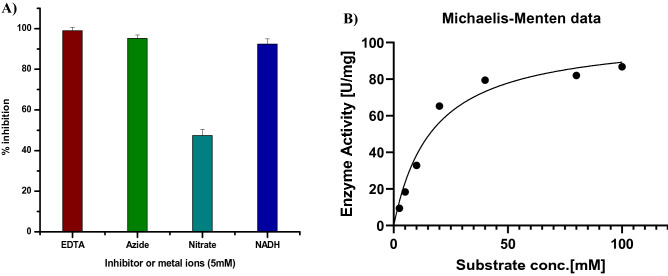
Characterization of NO production by the NirK protein: (**A**) Percent inhibition of the NO production by inhibitors (EDTA, azide, nitrate and NADH) was calculated using the formula {(Control-Test)/ (Control-Blank)}*100, where control is without any inhibitor or metal ions and test is the treated one. (**B**) Kinetics of NO formation by purified NirK protein in presence of nitrite. NO synthesis was fluorometrically monitored in presence of different concentrations of nitrite (2.5 to 100 mM) in 50 mM HEPES buffer at pH 7 as well as fixed small quantity of the purified protein. K_m_ value of Mtb NirK for nitrite was determined by using Graphpad software. The plot was drawn by taking nitrite reductase activity with different nitrite concentrations (2.5 to 100 mM) at pH 7. The data is represented as the mean values obtained from triplicate experiments ± SD.

Further, the recombinant NirK protein showed a significant increase in activity (62.21 ± 1.55 RFU) in the presence of 10 mM nitrite compared to heat denatured protein (9.75 ± 2.36) when monitored using DAF2 dye under identical conditions (Fig. 4C). The results indicated that the recombinant NirK protein has converted nitrite to NO (*P* < 0.01). As the *nirK* gene product represents nitrite reductase activity, we optimized the condition for the enzyme activity with respect to buffer, pH, temperature, substrates and possible co-factor/s requirements under in vitro conditions. The enzyme showed consistently better signal to noise ratio as well as lowest blank reading in 50 mM HEPES buffer compared to Tris HCl, sodium phosphate and potassium phosphate buffers at pH 7.0 respectively (Fig. S4A). So, the optimum pH of the recombinant NirK protein was found at 7.0 (Fig. S4B) and the optimum temperature detected at 37 °C (Fig. S4C). The tungstate and *iNOS* inhibitor 1400 W did not show any effect on nitrite reductase activity of the recombinant NirK protein (Fig. S4D,E), whereas NADH, EDTA, nitrate and azide at a final concentration of 5 mM (concentration used as per reported on other bacterial NirK enzymes) showed 92.34, 98.98, 47.40 and 95.16% of inhibition respectively under identical conditions (Fig. 5A, S4 D,E)^32^. The enzyme kinetics showed increased NO production with increased concentration of nitrite (2.5–100 mM) (Fig. 5B) attaining saturation level with K_m_ at 17.16 mM and K_cat_ at 0.1 × 10^3^ s^−1^ respectively. The catalytic efficiency of the purified recombinant NirK protein was calculated from K_cat_/K_m_ to obtain a value of 5.85S^−1^ mM^−1^. In addition, *MRA2164* gene was found to have 38% identity with *Pseudomonas aeruginosa,* 33.93% with *Nitrosomonas europaea* and 32% with *Escherichia coli* nitrite reductase (*nirK*), and *MRA2164* also have a close molecular mass of ~ 28 kDa, which is close to 32 kDa of *Nitrosomonas europaea, nirK* protein. Therefore, further study could elucidate which type of Cu center present in the Mtb *nirK* gene with its relevant significance.

### Expression of *MRA 2164* gene in *KDnirK Mycobacterium tuberculosis*

**Figure 6:**
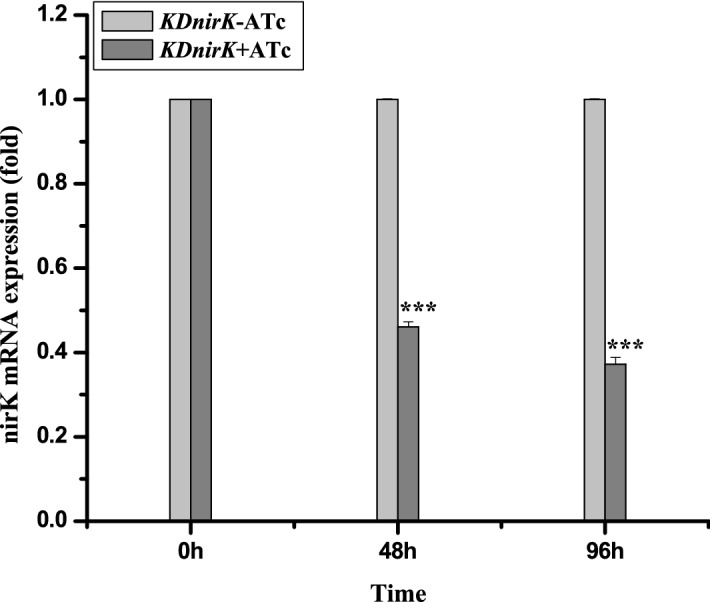
Level of mRNA of *MRA2164* gene expressed in ATc induces *KDnirK Mycobacterium tuberculosis*: Relative quantification was done by using SYBR green based qPCR kit and fold change were calculated for mRNA obtained from ATc induced *KDnirK* Mtb cells of 0 h, 48 h and 96 h (gray color). *SigA* gene was used as a housekeeping reference. The light gray color represents the fold change obtained from uninduced *KDnirK* Mtb cells as control. The data represented as the mean of three experiments ± SD.

The expression *MRA 2164* gene in *KDnirK* and *KDnirK* treated ATc *Mycobacterium tuberculosis* strain was analyzed by estimating their respective mRNA levels using q-PCR technique. It was observed that the knock down of the *MRA2164* gene in *KDnirK* treated ATc was down regulated to 0.46 fold at 48 h and 0.37 fold at 98 h compared to *KDnirK* without ATc treatment (Fig. 6). This has clearly established that a significant extent of down regulation in the expressions of *MRA 2164* gene happens in ATc-induced *KDnirK* cultures of Mtb strain.

### NO synthesis in *KDnirK* clone of *Mycobacterium tuberculosis*

**Figure 7:**
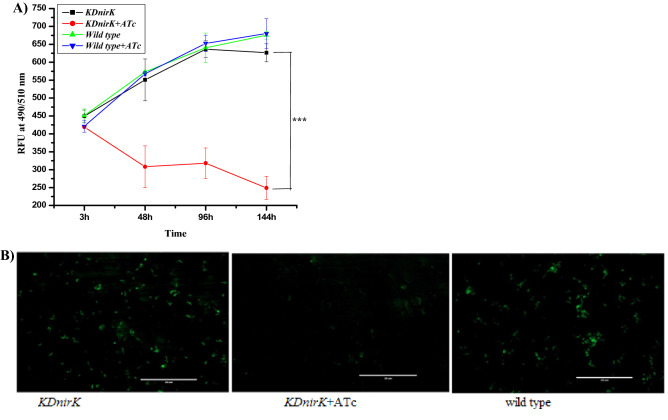
NO synthesis in *nirK* knock down clone of *Mycobacterium tuberculosis*: (**A**) Relative fluorescence units measured at 490/510 nm and (**B**) Fluorescence microscopy images captured of the *KDnirK* strain of Mtb bacilli in presence or absence ATc along with wild type control (Scale bar 50 μm) as mention in Materials and Methods section. The data is representative of triplicate experiments ± SD.

The conditional knock down of *nirK* gene was used to check the involvement of NirK in the intracellular NO production in the bacilli by using *dCas9* with gRNA. The knock down clone was grown in the presence of kanamycin and hygromycin as selection markers. The exposure of this knock down clone to 10 mM of nitrite in the medium did not show any difference with the wild type cells (636 ± 25 RFU at 96 h and 640 ± 41 RFU at 96 h). However, exposure of knock down clone to the ATc significantly decreased endogenous NO production as 318 ± 42 RFU at 96 h and 248 ± 32 RFU at 144 h respectively whereas wild type control showed fluorescence value as 640 ± 41 RFU at 96 h and 675 ± 25RFU at 144 h respectively (Fig. 7A). In addition, fluorescence microscopic images also confirmed the significantly reduced level of fluorescence intensity in the induced *KDnirK* compared to control or non-induced knock down clone (Fig. 7B). Therefore, this result clearly indicated that NO synthesis was significantly reduced (2–threefold decreased) in ATc induced *KDnirK* compared to wild type control or without ATc (acting as a complemented strain for *nirK* gene) respectively (Fig. 7A), which confirms a direct involvement of the *nirK* in nitrite dependent NO synthesizing activity in Mtb bacilli.

### Effect of nitrite on the growth of *KDnirK* Mtb strain

**Figure 8:**
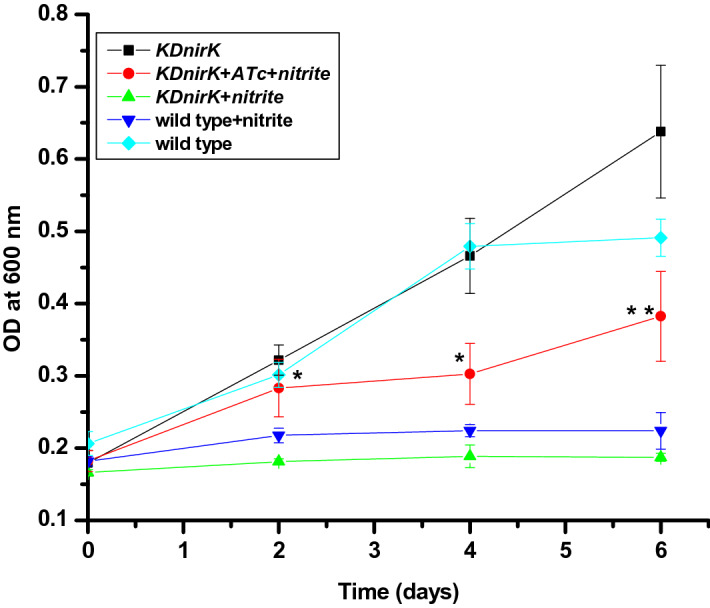
Effect of nitrite on growth of *Mycobacterium tuberculosis KDnirK* clone*:* Log phase cells of *KDnirK* (black filled square), *KDnirK* induced ATc with 10 mM of nitrite (black filled circle), *KDnirK* cells with 10 mM of nitrite without ATc (black filled triangle), wild type treated with 10 mM of nitrite (black filled inverted triangle) and wild type (black filled daimond) Mtb culture were incubated at 37 °C. The growth kinetics was monitored by measuring OD_600_ nm at different time point. The data represented as mean of two independent experimental results with (± SEM). Asterisks (*and **) indicating the significance difference between *KDnirK* induced cells with nitrite treated to *KDnirK* nitrite strain. Model 1: Synthesis of NO and its role in infected *Mycobacterium tuberculosis* NO and superoxide are produced from infected/Activated macrophage by *iNOS* and NADH oxidase (*Nox*) respectively. The reaction between NO and superoxide form peroxynitrite which is subsequently converted to nitrate and passively move ↑ inside phagosome. Under hypoxic condition, this nitrate further transport inside Mtb cells and utilized by *narG* to form nitrite. This increased nitrite facilitates further the entry of nitrate inside the bacillus through narK2 antiport while *nirK* reduces nitrite to NO as a mechanism of maintaining long-term latency inside human lungs.

Growth is the important characteristic features of the active state of mycobacteria. In our earlier report we have seen that nitrite induces VBNC state by inhibiting the growth of Mtb cells^24^. In order to correlate the effects of varying NO level on the development of non-/replicating status of Mtb bacilli when the growth of *KDnirK* Mtb strain was measured in presence of nitrite under conditions of w/o antibiotic induction (Fig. 8). The two independent experimental results clearly showed that in ATc induced *KDnirK* cells grow even in the presence of nitrite (10 mM) whereas the growth was inhibited when the culture is not induced by the antibiotic^24^. This clearly proved that knockdown has successfully been executed on the expression of *nirK* and the role of the gene is observed in converting nitrite to NO within the cell. The limitation of using this KD strain lies in the fact that the strain is difficult to maintain under constant ATc pressure within ex vivo and in vivo environments because of its stability issues. Although, the growth of ATc induced *KDnirK* culture was not as per with *KDnirK* in absence of nitrite but it was significantly (*P* < 0.03) different from nitrite treated *KDnirK* culture without ATc. This clearly indicated that NO, nitrite and *nirK* together play an important role in developing non-.replicative status of Mtb.

## Discussion

Nitrite is unique among the nitrogen oxides (NO_2_, NO, N_2_O) because of its redox position found between oxidative (NO_2_ radical) and reductive (NO radical) states^33^. Last few decades, a lot of data has been accumulated regarding nitrogen metabolism in Mtb and their role in the pathogenicity of the organism^15,17,18^. It was also noted that a significant extent of NO is released through exhaled air from inflammated lungs of infected patients which strongly suggest that activated alveolar macrophages are probably the major source of all RNI^34,35^. The involvement of nitrogen oxides in the intracellular life of Mtb cells was validated by reduced infectivity of Mtb in *iNOS* knocked out mutant macrophages^35^. Earlier studies have also detected the presence of nitrate and peroxynitrite within the hypoxic environment of granulomas in human lungs, are an oxidized product of NO from the activated alveolar macrophage cells^12,13^. The presence of nitrite in the lungs could be envisaged from the utilization of nitrate as respiratory substrate under hypoxic conditions by Mtb cells^16^. Therefore, our results clearly indicated that exposure to nitrite (> 5 mM), produces NO in Mtb which is capable of transforming the active stage cells to non-replicating ones (Fig. 1)^11^. However, the development of latency or VBNC cells could be dependent on the concentration and time of exposure to nitrite (Fig. 1).

Furthermore, the identification of the gene (*MRA2164)* with CDs specific to nitrite dependent NO producing enzyme, as a ~ 26kDa protein in Mtb indicated about the presence of functional NirK enzyme in the pathogen (Fig. 2A,B). Subsequently, extensive characterization of the cloned and purified *MRA2164* protein under different experimental conditions has clearly showed that it is functionally very similar to NirK protein known to be present in other bacteria (Figs. 4, 5, S4)^36–38^. The Km of this enzyme was found to be ~ 17 mM, which justifies the level of NO produced at > 5 mM of nitrite in the medium (Figs. 1, 5).

The roles of the Mtb NirK (*MRA2164*) gene was further confirmed from a significantly reduced level of NO production in the presence of rutin hydrate and knock down clone (confirmed by significantly reduced level of mRNA expression in the presence of ATc) of it under identical conditions as well as the reduced effect of nitrite on the transformation from active to the non-replicative stage (Figs. 6, 7, 8). Overall, our results clearly demonstrated that the Mtb gene *MRA2164* function as NirK enzyme by synthesizing NO from nitrite under in vitro conditions as well as with in the cell (as NO production was not observed in the presence of nitrate and arginine as a substrate) (Fig. S5).

In bacteria, the endogenously formed NO is controlled by the nitrogen oxide cycle and acts as a signaling molecule for morphological differentiation (e.g. spore formation in *Streptomyces coelicolor*)^39^, and infection^40,41^ However, there is no report about NO acts as an inducer for dormancy in these cells. In the case of Mtb, NO was earlier shown to have a cidal effect on Mtb in the murine model while in human, the survival of Mtb bacilli in the presence of NO remained controversial so far^35^. However, earlier report coupled with results obtained from the present study suggested that NO induces viable but non-cultivable (VBNC) state in actively growing Mtb cells using nitrite as substrate. Our data also suggests that Mtb *nirK* expression is up regulated by nitrite to attain a peak level within 24 h depending on the concentration of nitrite in the medium (Figs. 3, 5). Further study is required to carry out to understand the sequence and activities of regulatory genes in dormancy development. As an immediate stable source of NO to the site of interaction probably maintained within human lungs for prolong period to keep Mtb cell remain in a latent state. So, the accumulated reports along with the present study clearly indicates that nitrite exposure could develop two different conditions (1) pro-growth at lower concentrations (< 5 mM) using the assimilatory pathway of nitrogen metabolism and (2) pro-latency at higher concentrations.

In summary, the intracellular production of NO by *MRA2164* gene product in Mtb is completely a novel and crucial finding to explain the attainment of long term latent TB in human lungs to better understand the pathogenesis of latent TB and its management. The purpose of the study is to precisely understand the effect of the exposure of different levels of nitrite on the bacilli. We preferred to use 10 mM nitrite for our study because of the observed toxicity against Mtb at higher concentration and at lower concentrations; the use of nitrite is restricted probably by the some unknown intracellular effector molecule/s for NO production. This in vitro experiment has provided the scope to help understand the bacterial system in absence of complex in vivo conditions. Therefore, these findings could potentially help elucidate the mechanism of RNI regulated survival of Mtb under in vivo condition and future course of anti-TB treatment.

## Materials and methods

### Chemicals and reagents

All chemicals, antibiotics were purchased from Sigma-Aldrich, the USA, otherwise mentioned. Dubos medium was procured from DIFCO, USA. T4 DNA ligase and restriction/ DNA modifying enzymes were obtained from New England, Biolabs. Luria–Bertani (LB) broth was, purchased from Hi-media, India. DAF2 was purchased from Everon life sciences. Molecular weight markers were bought from BioRad.

### Bacterial strains and culture conditions

*M. tuberculosis* H37Ra (ATCC 25,177) was obtained from the Microbial Type Culture Collection (MTCC) Chandigarh, India. The pET28a (Invitrogen) was used as a cloning and expression vector. *E. coli* DH5α and *E. coli* Rosetta-gami (Novagen) were used as cloning and expression hosts, respectively. Plasmids pRH2502 & pRH2521 were gifted from (Addgene plasmid #84,379, #84,380) in *Escherichia coli* DH5*α* strain, which was cultured in Luria–Bertani broth with kanamycin (50 µg/mL) and hygromycin (100 µg/mL) respectively. Mtb H37Ra was grown in Dubos medium containing 5% glycerol and 10% ADC (albumin, dextrose, and catalase supplement) at 37 °C in a shaker incubator (Thermo Electron Model No.131 481; Thermo Electron Corp., Marietta, OH) at 150 rpm. The stock culture was maintained at − 70 °C and subcultured once in the liquid medium before inoculation to an experimental culture.

### Detection of intracellular nitric oxide of *Mycobacterium tuberculosis*

A NO detection kit (FCANOS1, Sigma Aldrich) was used to detect NO within Mtb cells by following the manufacture’s instruction manual. Briefly, 1.5 mL of Mtb log-phase culture (0.30 OD_600_) was taken in the 24-well plate, supplemented with 10 mM of nitrite to induce a viable but non cultivable dormancy state in actively replicative bacilli by following an earlier protocol^24^. The plate was then incubated at 37 °C in a CO_2_ incubator. After 24 h, an aliquot of 200 μL of culture was transferred to 96 well black plate, to which 100 mM phosphate buffer (pH 7.2) containing DAF-2DA (2.5 μM) was added to make a final volume 300 μL, and incubated for 2 h at 37 °C in the dark. Fluorescence was measured at excitation 490 nm and emission 510 nm by using a multimode plate reader (Model Spectramax M5e, Molecular Devices, USA). The medium containing nitrite (10 mM) was used as a culture blank.

For microscopic fluorescence studies, after 2 h incubation of Mtb with DAF-2DA, cells were washed twice with PBS by centrifuging at 5,000 rpm for 5 min. The pellet was re-suspended in 100 μL of PBS. The smear was prepared on a grease-free glass slide, and the fluorescence images were captured using a fluorescence microscope with 60 × objectives (EVOS, Life Technology, Germany).

### Identify the probable NO forming genes in *Mycobacterium tuberculosis*

Nitrite reductase (NO forming) gene sequences from 27 different bacteria, which are evolutionarily near to Mtb*,* were collected from the KEGG pathway database (http://www.genome.jp/kegg/pathway.html). Conserved sequences for nitrite reductase (NO forming) genes from different bacterial species were determined by using ClustalW2 software (http://www.ebi.ac.uk/ Tools/msa/clustalo/). Conserved sequences obtained were further analyzed for the presence of conserved domains (CDs) by using CD-search software (https://www.ncbi.nlm.nih.gov/ Structure/cdd/wrpsb.cgi). All CDs of copper oxidase were searched in the Mtb genome database. In bacteria, two types of nitrite reduction enzymes have been discovered with distinct molecular structures and prosthetic groups *i.e.,* cytochrome cd1heme and copper nitrite reductase^42,43^. The copper-containing nitrite reductase (CuNiR) is most abundant and was found in about one-fourth of the denitrifying bacteria^42^. Interestingly, copper-containing nitrite reductase of *Pseudomonas aeroginosa* was found to produce NO under aerobic conditions using nitrite as a substrate^44^.

### Isolation and quantification of RNA from *Mycobacterium tuberculosis*

RNA was isolated from Mtb cells treated with or without 10 mM of nitrite using a spheroplast solution followed by Trizol based method^45^. Briefly, the spheroplast solution was aseptically added to the nitrite treated Mtb cultures on 1, 3, and 6 days of incubation. Cells were harvested, and total RNA was isolated. 1 μg of total RNA was reverse transcribed for the preparation of cDNA by using a single-strand synthesis kit (Sigma Aldrich) as per manufacture’s instruction. The qPCR was performed by using Quantitative SYBR Green PCR kit as per manufacturer’s instruction. The qPCR reaction was run at 95 °C for 5 min, followed by 40 cycles of 95 °C for 30 s, annealing temperature 58 °C for 30 s, and 72 °C for 30 s (PikoReal96, Thermo Scientific). The gene-specific primers were used for *MRA2164* (P1 & P2) and *MRA0854* (P3 & P4), whereas *SigA* was used as a housekeeping gene (Table 3). We have used TTEST for all statistical analysis.

**Table 3:** Primer used for this study.

Primer name	Sequence details (5ʹ–3ʹ)	Annealing temp. (°C)
P1	ACTGGGAACGTGAGTGTTCG	58
P2	GGTGTCATCGAGTGCCGTAT	58
P3	CACCATGGCCAAGTACGA	58
P4	TGAAAGGTATGGCCGTGTAG	58
P5	GCCGGATCCCATCATCATCATCATCAT TT GCT CGC CAG TAC GCG	55
P6	GCCAAGCTTTCATTCCATCCACACCAACGACGC	55
P7	GGGAGGTCGACCAGCCGCGCAATA	55
P8	AAACTATTGCGCGGCTGGTCGACC	55

### Cloning of *MRA2164* gene from *Mycobacterium tuberculosis* in pET 28a vector

The genomic DNA was isolated from mycobacterial cells, as described earlier^46^. The *MRA2164* gene sequence of Mtb obtained from the NCBI database was amplified by the PCR using gene-specific primers (P5 & P6 in Table 3) containing *BamHI* (Forward) & *HindIII* (Reverse) restriction sites. The amplified PCR product of *MRA2164* was purified and digested with *BamHI* and *HindIII* restriction enzymes along with the pET28a vector. A digested product was then ligated into expression vector pET28a and transformed into *E. coli* DH5α cells using a standard protocol. The *MRA2164* gene transformed colonies were screened by colony PCR using the *MRA2164* specific primers described above (P5 & P6 in Table 3). The positive clone was isolated and confirmed by restriction digestion and sequencing.

### Protein expression and purification

The pET28a- *MRA2164* clone was transformed into the *E. coli* Rosetta-gami strain. The transformed cells were grown in LB broth with kanamycin (50 µg/mL) at 37 °C up to 0.5 of OD_600nm_ and induced with 0.25 mM IPTG for 3 h. After induction, cells were pelleted, lysed in buffer containing 50 mM Tris–HCL, 300 mM NaCl, 0.1% Triton X-100, 1% protease inhibitor cocktail, 10% glycerol pH 7.5, followed by sonication with 40 kHz for 30 s on and 45 s off cycles for 10 min. The recombinant protein was recovered as inclusion bodies in pellet by centrifugation at 10,000 rpm for 45 min. The pellet was treated with 8 M urea dissolved in 50 mM Tris–HCl buffer with continuous stirring for 1 h at room temperature. The supernatant was collected by centrifugation at 14,000 rpm for 1 h at room temperature. Then, the supernatant was subjected to the NI–NTA His-tag column. The eluted protein further dialyzed in 50 mM Tris–HCL pH 7.0, 1 mM CuSO_4_ and 10% glycerol was obtained in the refolded form. The purity of the protein was assessed by SDS-PAGE and mass identification after trypsin in-gel digestion of the protein analysis using SYNAPT LC–MS.

### Isolation and quantification of RNA from *KDnirK Mycobacterium tuberculosis*

RNA was isolated from *KDnirK* Mtb cells treated with or without ATc using a spheroplast solution followed by a Trizol based method used earlier^45^. cDNA was synthesized from wild type, 2 days and 4 days ATc treated *KDnirK* and without treated *KDnirK* Mtb strain and qPCR was performed by using SYBR green PCR kit as described in earlier methodology section.

### Knock down of *MRA2164 (KDnirK)* gene in *Mycobacterium tuberculosis*

The knock down of Mtb for the *MRA2164* gene was developed as a conditional mutant by using CRISPR/dCas9 based method optimized by following an established protocol^47^. Briefly, the selected primers (P7 & P8 in Table 3) were used as sgRNA for cloning into the pRH2521 vector (Fig. S1)^48^. Then, a positive sgRNA clone was screened, transformed into competent dCas9 Mtb, and colonies were obtained after 4 weeks of incubation on the Middlebrook7H9 agar medium containing kanamycin (25 µg/mL) and hygromycin (50 µg/mL) as selection markers. The Mtb cells expressing dcas9 with sgRNAs *MRA2164* (*KDnirK*) were maintained in Dubos broth containing kanamycin (25 µg/mL) and hygromycin (50 µg/mL). The conditional knock down of the *KDnirK* strain was induced by Anhydrotetracyclin (ATc) (200 ng/mL). Periodically NO detection and fluorescence microscopic studies were performed for *KDnirK* strain by using DAF 2DA dye as described above.

### Characterization of purified recombinant NirK enzyme

The NO synthesizing activity of purified recombinant NirK protein was determined by using DAF2 as a probe by following a modified method described earlier^49^. Briefly, 1 µg of protein transferred in 96 black well plate in a final volume of 150 μL 50 mM HEPES buffer pH 7.0 containing dye DAF2 (5 nM) and incubated for 2 h at 37 °C temperature. The fluorescence was measured at excitation 490 nm and emission 510 nm wavelength, respectively. The Km and Vmax values were calculated using Graphpad Prism software (Graphpad, San Diego, CA) of a non-linear regression plot using the Michaelis–Menten equation.

### Growth kinetics of *KDnirK* Mtb strain in the presence of nitrite

The conditional mutant of the *KDnirK* strain was induced by the addition of ATc (200 ng/mL). After 24 h treatment of ATc, culture aliquot transferred in 24 well plate, to which 10 mM of nitrite was added. The OD _600_ nm was measured at different time points. ATc blank culture without nitrite considered as a negative control for growth. ATc concentration was maintained with fresh addition after every 48 h. Wild type culture with 10 mM of nitrite and without nitrite with the same OD _600_ considered as control.

## Supplementary Information

Supplementary Information.
