# Supplementary material for: Novel gene similar to nitrite reductase (NO forming) plays potentially important role in the latency of tuberculosis
Source: Sci Rep. 2021 Oct 6;11:19813. doi: 10.1038/s41598-021-99346-1 (PMC8494734; doi:10.1038/s41598-021-99346-1)

**Supplementary Files**

**Novel gene similar to nitrite reductase (NO forming) plays potentially important role in the latency of tuberculosis**

**Authors: Sonia Agrawal ^1, 2^, Suwarna Gample ^1, 2^, Amar Yeware ^1, 2^, Dhiman Sarkar ^1, 2,^ ***

**Author’s Affiliation, Address:**

^1^ CSIR-National Chemical Laboratory, Organic Chemistry Division, Dr. Homi Bhabha Road, Pune-411008, Maharashtra, India.

^2^ Academy of Scientific and Innovative Research (AcSIR), Ghaziabad, 201002, India.

Tel: +91-20-25902860 (O), Fax: + 91-20- 25902624.

**Figure S1:** **Vector map of *KDnirK*** Using SnapGene vector map were prepared. Plasmids pRH2502 having *dcas9* expression gene and pRH2521 vector having guide RNA expression gene. Plasmid pRH2521 sg RNA vector electroporated into Mtb pRH2502 combine after treating tetracycline reduces the target gene at RNA expression.


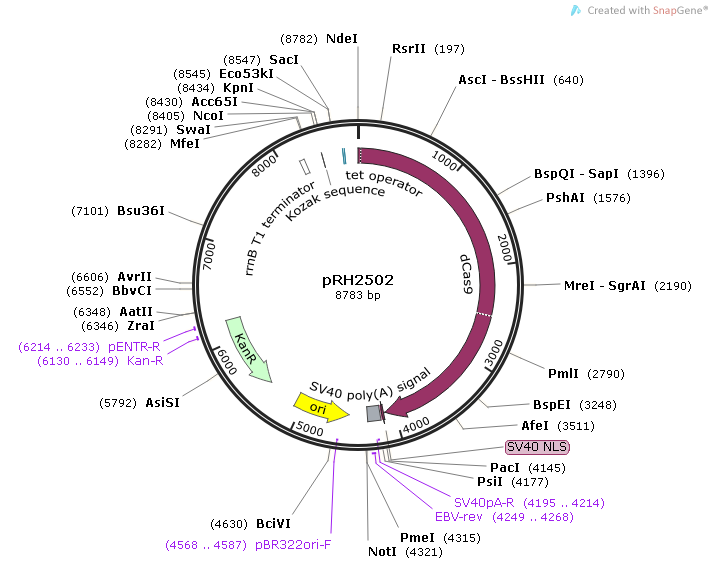

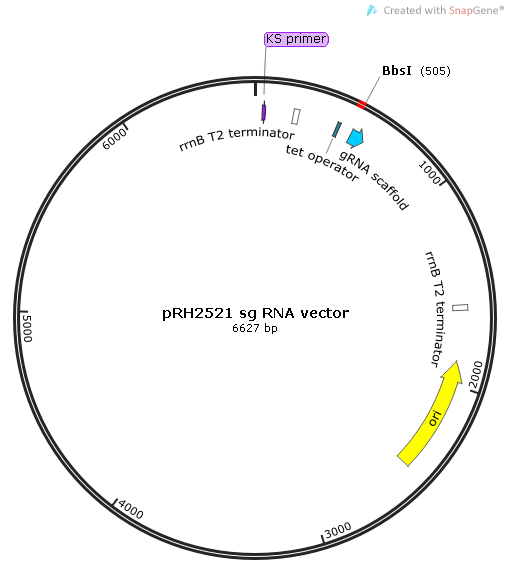


**Figure S2: Endogenous NO production** **in *E.coli* & *M. smegmatis*:** Log phase of *E.coli* & *M. smegmatis* cells were treated with nitrite (10mM) and without nitrite , and NO was detected by using DAF 2DA dye. More details are provided in “Materials and methods” section. The data shown is representative as mean of three independent experiments ±SD.

**Figure S3:** **Cloning of *MRA2164* gene from Mtb in pET 28a vector:** Restriction analysis to confirmed the recombinant *MRA2164* in peT28a vector by 1) double digestion using *NdeI* & *HindIII* restriction enzyme 2) undigested vector, M-Marker


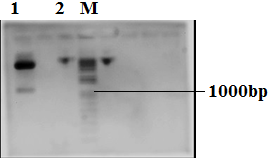


**Figure S4 A-E: Characterization of *MRA2164* protein for NO synthesis activity A)** NO synthesis activity in a different buffer such as TRIS, HEPES, Sodium phosphate and Potassium phosphate **B**) The effect of pH on *MRA2164* protein activity. The activity was assayed at 37 ^o^C in HEPES buffer containing a pH range of 6.0-8.0. **C)** Effect of temperature. The activity was performed at pH 7.0 in HEPES buffer at 22-42 ^o^C. The fluorescence was measured after 2h incubation at 22, 30, 37 and 42 ^o^C. **D)** Effect of Cofactor or metal ions such as copper, zinc, ferric and NADH at a final concentration of 5mM. **E)** Effect of inhibitors EDTA, sodium azide, sodium tungstate, *iNOS* inhibitor and sodium nitrate at a final concentration of 5mm was used for NO detection. The results represented as the mean of three identical experiments ±SD.

1. **B)**

**C) D)**

**E)**

**Figure S5: NO detection in the presence of nitrite, arginine and nitrate as a substrate:** Log phase Mtb cells were treated with nitrite (10mM), L-arginine (10µL) and nitrate (10mM) and NO was detected as per manufacture’s instruction using DAF 2DA dye. More details are provided in “Materials and methods” section (P ≤ 0.002).


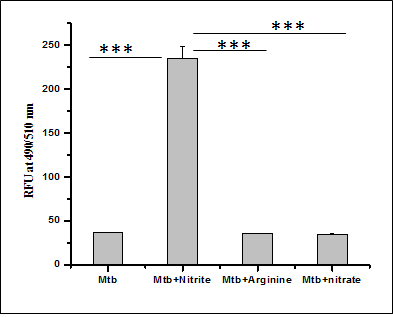

Supplement: Supplementary file 1 — Supplementary Information. [file 41598_2021_99346_MOESM1_ESM.docx]
